# 基于镜像酶正交酶切的蛋白质复合物规模化精准分析新方法

**DOI:** 10.3724/SP.J.1123.2021.06010

**Published:** 2022-03-08

**Authors:** Ruonan HAN, Lili ZHAO, Yuxin AN, Zhen LIANG, Qun ZHAO, Lihua ZHANG, Yukui ZHANG

**Affiliations:** 1.大连理工大学, 张大煜学院, 辽宁 大连 116024; 1. Zhang Dayu School of Chemistry, Dalian University of Technology, Dalian 116024, China; 2.中国科学院大连化学物理研究所, 中国科学院分离分析化学重点实验室, 辽宁 大连 116023; 2. CAS Key Laboratory of Separation Sciences for Analytical Chemistry, Dalian Institute of Chemical Physics, Chinese Academy of Sciences, Dalian 116023, China; 3.中国科学院大学, 北京 100049; 3. University of Chinese Academy of Sciences, Beijing 100049, China

**Keywords:** 化学交联质谱, 多酶酶切, 蛋白质复合物, 镜像酶, chemical cross-linking mass spectrometry technology (CXMS), multi-protease digestion, protein complex, mirror protease

## Abstract

蛋白质主要以复合物的形式参与各项生命活动。化学交联质谱(CXMS)技术作为近年来新兴的蛋白质复合物解析技术,不仅可实现蛋白质复合物规模化解析,而且普遍适用于任意相对分子质量和纯度的蛋白质复合物样品,因此已成为X-射线晶体衍射技术、冷冻电镜技术等蛋白质复合物解析经典技术的重要补充。目前,CXMS主要采用胰蛋白酶将交联后的蛋白质复合物进行酶切,并进行质谱鉴定。然而鉴定到的交联肽段谱图中b/y特征碎片离子的鉴定数目不足,且因肽段序列匹配连续性较差导致谱图可信度相对较低,影响了蛋白质复合物交联位点的鉴定精准度。该文基于镜像酶正交切割的互补特性,采用胰蛋白酶镜像酶与胰蛋白酶联合酶切的方法,对交联后的蛋白质复合物分别进行酶切,再利用液相色谱-质谱联用技术对酶解产生的交联肽段进行鉴定,进而实现蛋白质复合物的组成、相互作用和结构位点距离约束的解析。对牛血清白蛋白和大肠杆菌全蛋白的交联肽段的分析结果表明,该方法通过增加交联肽段谱图中特征碎裂离子的数目和连续性,鉴定到的大肠杆菌全蛋白的交联位点,较单一胰蛋白酶酶切,提高了16%。因此,镜像酶正交酶切策略能有效提高交联肽段的鉴定准确度和覆盖度,有望为实现规模化的蛋白质复合物精准解析提供新思路。

## Abstract (en)

Protein complexes are involved in a variety of biological activities. Accurate and comprehensive characterization of the structures and interactions of protein complexes is crucial in determining their biological functions. Chemical cross-linking coupled with mass spectrometry (CXMS) is an emerging investigative technique for protein complexes. CXMS enables the sensitive high-throughput analysis of protein complexes without the requirements of molecular weight and purification. These attributes have spurred the increased use of CXMS for the structure and interaction characterization of purified protein complexes and complicated cell lysate samples. CXMS utilizes chemical cross-linking reagents to covalently connect two reactive amino acids in or between proteins that are spatially close to each other. Subsequently, the cross-linked proteins are digested into cross-linked peptides, followed by LC-MS/MS analysis, as well as database searching to provide cross-linking information for the composition, interaction, and structural site distance restrictions of protein complex identification. Therefore, identification of cross-linked sites has a decisive influence on the characterization of protein complexes. This identification is limited by the unsatisfactory quality of the cross-linked peptide spectrum. Insufficient b/y fragment ions and poor continuity of amino acid sequence matching lead to low coverage and accuracy of cross-linked site identification. Based on the complementary feature of mirror-cutting digestion, an orthogonal digestion strategy based on LysargiNase combined with trypsin was developed in this study. Trypsin is the most commonly utilized digestion enzyme in proteomics, with extremely high enzyme activity and specificity. Trypsin generates C-terminally charged peptides after lysine (K) and arginine (R). LysargiNase is a mirror protease complementary to trypsin that cleaves before the K and R residues. This generates peptides with an N-terminal positively-charged residue. Owing to the different physical and chemical micro-environments of the cross-linked peptides digested by LysargiNase and trypsin, the behavior of their detection ability in MS analysis is diverse. Using the orthogonal digestion strategy, both simple and complicated cross-linked samples were analyzed in this study. For the analysis of bovine serum albumin (BSA), 291 pairs of non-redundant cross-linked sites were obtained, of which 216 pairs of cross-linked sites were provided by trypsin digestion, whereas 75 pairs of cross-linked sites were exclusively supplied by LysargiNase digestion. Except for the 35% increase in the number of identified cross-linked sites, 32% of the spectra of the commonly identified cross-linked peptides have better quality with more b-type fragment ions and consecutive sequence matching. Furthermore, for the *Escherichia coli* sample, 726 pairs of cross-linked sites were obtained in total, among which, 624 and 274 pairs were identified from trypsin and LysargiNase digestion, respectively. LysargiNase digestion yielded 120 individual cross-linked sites, which resulted in a 16% increase in single trypsin digestion. Consistent with the BSA sample, the quality was improved in 35% of the spectra of commonly identified cross-linked peptides. Corresponding to the identified cross-linked peptides, 242 structural constraints with 607 pairs of intra-cross-linked sites and 29 sets of protein-protein interactions with 119 pairs of inter-cross-linked sites were obtained. The collective results demonstrated that, mirror cutting-assisted orthogonal digestion strategy could significantly increase the number of identified fragment ions and amino acid sequences matching the continuity of the spectra by contributing b-and y-type ions, respectively. This improved the accuracy and coverage of cross-linked peptide identification. The findings additionally demonstrate the superiority of our method in the accurate identification of the cross-linked peptide spectra and the increased number of identified cross-linked sites. In a word, this method is expected to provide new insights for the large-scale and highly accurate characterization of protein complexes.

蛋白质作为生命活动的执行者,通过自身结构的动态改变,以及与其他蛋白质相互作用组装为蛋白质复合物,调控各种生物学过程。因此,如何实现蛋白质复合物的精准解析已成为当前生命科学的研究热点。化学交联结合质谱(CXMS)技术作为蛋白质复合物解析的新兴技术,利用化学交联剂将空间距离足够接近的蛋白质分子内或分子间的氨基酸残基以共价键连接起来,再利用液相色谱-质谱联用对交联肽段进行鉴定,实现蛋白质复合物的组成、界面和相互作用位点的解析。该技术具有分析通量高、灵敏度高、可提供蛋白质间相互作用的界面信息、普遍适用于不同种类和复杂程度的生物样品等优势,已成为X射线晶体衍射^[1,2]^、低温冷冻电镜^[3,4]^、免疫共沉淀^[5,6]^等蛋白质复合物研究技术的重要补充。

化学交联位点的鉴定覆盖度和准确度决定着该技术对于蛋白质复合物结构的解析能力。目前,为了实现蛋白质复合物的高覆盖度交联,研究人员发展了可用于共价交联赖氨酸(K)的氨基、谷氨酸(E)/天冬氨酸(N)的羧基^[7]^、精氨酸(R)的胍基^[8]^以及半胱氨酸(C)的巯基^[9]^等多种活性基团的新型交联剂。进而,为了提高低丰度交联肽段的鉴定灵敏度,体积排阻色谱法^[10]^、强阳离子交换色谱法^[11]^,及亲和基团富集策略被提出用于交联肽段的高选择性富集,如可富集型化学可断裂交联剂——Leiker^[12]^,与不具备富集功能的交联剂相比,通过亲和富集可以将交联位点鉴定数目提高4倍以上。此外,为了提高质谱对交联肽段的鉴定能力,解决由于交联肽段长度过长以及多级交联导致的鉴定困难的问题,Leitner等^[10]^通过对8种标准蛋白质及20S蛋白酶体的交联实验,初步证明多酶酶切方法能够有效提高交联位点的鉴定数目;Zhao等^[13]^提出了基于“smart cutter”多种酶联用的原位顺序酶解策略,将大肠杆菌中蛋白质复合物交联位点的鉴定数目相对单一胰蛋白酶(trypsin)酶切提高了54%。在交联数据分析方面,为提高交联位点的解析精度和效率,一系列的CXMS数据解析软件被开发,包括xQuest^[14]^、MassMatrix^[15]^和pLink^[16,17]^等。其中pLink在充分利用全部碎片离子的基础上提出了粗细两步打分策略用来缩减候选肽段规模,使鉴定速度得到了提高^[18]^。

上述技术虽然在很大程度上推动了基于CXMS的蛋白质复合物的解析覆盖度,但是交联肽段在质谱中存在多处碎裂,碎片离子形式多样,且存在交联基团与肽段共价连接的特异离子,因此交联肽段二级谱图的碎片离子比常规肽段的谱图在种类和数目上更为复杂^[16]^,会造成交联肽段鉴定谱图中碎片离子的匹配数目较少和肽段序列匹配连续性较差,导致谱图可信度较低,影响蛋白质复合物交联位点的鉴定精准度。

胰蛋白酶镜像酶(LysargiNase)的酶切位点与胰蛋白酶互为镜像,可特异地切割赖氨酸和精氨酸的N端。由于LysargiNase的N端酶切特点,电荷主要分布在交联肽段的N端,在碰撞诱导裂解(CID)和高能诱导裂解(HCD)模式下产生以b离子为主的碎片离子,与胰蛋白酶酶切肽段以y离子为主的碎片离子互为镜像补充,为胰蛋白酶酶解肽段在质谱鉴定中b离子缺失严重的问题提供了很好的解决办法^[19]^。由于具有较高的酶切特异性和酶活性,镜像酶已经成功地应用于蛋白质C末端蛋白质组鉴定^[20]^、磷酸化蛋白质组研究^[21]^、甲基化蛋白质组鉴定等^[22]^方面,然而在CXMS中的应用仍未见报道。

为进一步提高对蛋白质复合物结构及相互作用位点的解析能力,本文发展了LysargiNase与胰蛋白酶联合酶切的方法,基于镜像酶正交切割的互补特性,通过产生赖氨酸及精氨酸镜像分布的交联肽段,以增加特征碎片离子数量和肽段匹配连续性,从而提升交联肽段的谱图鉴定质量,达到提高交联位点的鉴定覆盖度和准确度的目的。通过分别对牛血清白蛋白及大肠杆菌全蛋白样品的交联位点鉴定结果的考察,评价该策略对单一蛋白样品和复杂细胞裂解液样品蛋白质复合物表征的应用潜力。

## 1 实验部分

### 1.1 仪器、试剂与材料

蛋白酶抑制剂(cocktail)、二硫苏糖醇(DTT)、三(2-羧乙基)膦(TCEP)、牛血清白蛋白(BSA)均购于美国Sigma-Aldrich公司;二(磺基琥珀酰亚胺)辛二酸酯(BS^3^)购于美国Thermo Fisher公司;胰蛋白酶、胰蛋白酶镜像酶购于中国华利世公司;BCA试剂盒购自中国碧云天公司;色谱纯乙腈(ACN)购自德国Merck公司;所有实验用水均经过美国Millipore公司购买的Milli-Q系统纯化;其他试剂均为分析纯。用于质谱分析的分离柱(15 cm, 150 μm i.d., 365 μm o.d.)中装有购于德国Dr. Maisch公司的ReproSil-Pur C18-AQ颗粒(颗粒大小为1.9 μm;孔径为12 nm)。熔融石英毛细管(150 μm i.d., 365 μm o.d.)购于中国Sino Sumtech公司;Venusil XBP C18硅胶填料(5 μm, 12 nm)购于中国博纳艾杰尔公司;Empore disk C18固相萃取膜片购于美国3M公司;超声破碎仪购自美国Cole-Parmer公司;真空浓缩仪、超微量分光光度计(Nanodrop one)、Easy-nano LC 1000系统、Q-Exactive质谱仪、Easy-nano LC 1200系统及Orbitrap Fusion Lumos质谱仪均购自美国Thermo Fisher公司。

### 1.2 实验方案

1.2.1 蛋白质样品制备

称取牛血清白蛋白粉末,以20 mmol/L 4-(2-羟乙基)-1-哌嗪乙磺酸(HEPES, pH 7.5)作为缓冲体系,配制0.1 mmol/L牛血清白蛋白溶液。

大肠杆菌细胞(种属K12)在37 ℃下采用Luria-Bertani(LB)培养基培养24 h,然后于4 ℃以4000 g离心2 min,收集细胞沉淀。细胞沉淀采用磷酸盐缓冲液(PBS)清洗3遍后,悬浮于细胞裂解液(含20 mmol/L HEPES和1%(v/v)蛋白酶抑制剂)中,冰浴超声破碎180 s(30%能量,10 s开,10 s关)。匀浆液于4 ℃以20000 g离心40 min,收集上清,采用BCA试剂盒测定所得蛋白质含量。稀释大肠杆菌蛋白裂解液至蛋白质含量为0.5 mg/mL。

1.2.2 化学交联样品制备

以20 mmol/L HEPES(pH 7.5)为溶剂配制浓度为20 mmol/L 的BS^3^交联剂母液;将交联剂母液加入牛血清白蛋白的缓冲溶液及大肠杆菌蛋白裂解液中,使交联剂的终浓度为1 mmol/L,在室温条件下反应15 min;通过添加终浓度为50 mmol/L的淬灭溶液NH_4_HCO_3_进行交联反应淬灭,并在室温下孵育15 min;在冰浴条件下,将交联样品逐渐滴入8倍体积的预冷丙酮中,于-20 ℃静置过夜;在4 ℃条件下,以16000 g转速离心,去除丙酮,然后将交联蛋白用预冷丙酮清洗2次,去除上清液后,于室温挥发掉残余的丙酮;以8 mol/L尿素溶液复溶蛋白质沉淀;将牛血清白蛋白交联样品以5 mmol/LTCEP作为还原剂,于25 ℃下反应1 h进行变性和还原;将大肠杆菌样品以5 mmol/LDTT作为还原剂,于25 ℃下反应1 h进行变性和还原,避免大肠杆菌蛋白在酸性条件下发生变性;添加终浓度为10 mmol/L的碘乙酰胺(IAA),在黑暗中,于室温下反应30 min;以50 mmol/LNH_4_HCO_3_稀释样品至尿素浓度为0.8 mol/L后,将样品均分为两份,一份以蛋白样品与蛋白酶的质量比呈50:1的比例加入胰蛋白酶,于37 ℃酶解过夜,另一份加入终浓度为20 mmol/L的CaCl_2_,以蛋白样品与蛋白酶的质量比呈20:1的比例加入LysargiNase,并在37 ℃温度下酶解过夜。

1.2.3 液相色谱-质谱鉴定及数据搜索

上述所有样品经过除盐,使用0.1%甲酸(FA)溶液复溶,用超微量分光光度计测定肽段浓度,进行反相高效色谱分离和质谱分析。

牛血清白蛋白样品采用Easy-nano LC 1000系统偶联Q-Exactive质谱仪平台进行质谱分析。流动相A: 2%(v/v)乙腈水溶液(含0.1%(v/v)FA);流动相B: 98%(v/v)乙腈水溶液(含0.1%(v/v)FA)。梯度洗脱程序:0~10 min, 2%B~7%B; 10~60 min, 7%B~23%B; 60~80 min, 23%B~40%B; 80~82 min, 40%B~80%B; 82~95 min, 80%B。Q-Exactive质谱仪采用数据依赖性模式,Full MS扫描在Orbitrap上实现,扫描范围为*m/z* 300~1800,分辨率为70000(*m/z*=200),自动增益控制(AGC)为3×10^6^,最大注入时间(IT)为60 ms,母离子分离窗口为*m/z* 2。MS/MS扫描的分辨率为17500(*m/z*=200),碎裂模式为HCD,归一化碰撞能量(NCE)为35%, MS2从*m/z* 110开始采集,MS2的AGC为5×10^4^, IT为60 ms,仅选择电荷值为3~7且强度高于1000的母离子进行碎裂,并将动态排除时间设置为20 s。每个样品分析3遍。

大肠杆菌样品采用EASY-nano LC 1200系统偶联Orbitrap Fusion Lumos三合一质谱仪平台进行质谱分析。流动相A: 0.1%(v/v)甲酸水溶液;流动相B: 80%(v/v)乙腈水溶液(含0.1%(v/v)FA)。梯度洗脱程序:0~28 min, 5%B~16%B; 28~58 min, 16%B~34%B; 58~77 min, 34%B~48%B; 77~78 min, 48%B~95%B; 78~85 min, 95%B。Orbitrap Fusion Lumos三合一质谱仪采用数据依赖性模式,Full MS扫描在Orbitrap上实现,扫描范围为*m/z* 350~1500,分辨率为60000(*m/z*=200), AGC为4×10^5^, IT为50 ms,母离子分离窗口为*m/z* 1.6。MS2扫描的分辨率为15000(*m/z*=200),碎裂模式为HCD, NCE为30%, MS2从*m/z* 110开始采集,MS2的AGC为5×10^4^, IT为60 ms。仅选择电荷值为3~7且强度高于2×10^4^的母离子进行碎裂,并将动态排除时间设置为20 s。每个样品分析3遍。

质谱数据文件(*.raw)采用pLink 2软件(2.3.9)对交联信息进行检索和鉴定。使用从UniProt于2019年4月27日下载的牛血清白蛋白序列和大肠杆菌序列,搜索参数如下:酶切方式为胰蛋白酶(酶切位置:K/R的C端)、LysargiNase(酶切位置:K/R的N端);漏切位点个数为3;一级扫描容忍(precursor tolerance)2.00×10^-5^;二级扫描容忍(fragment tolerance)2.00×10^-5^;每条肽段的质量范围为500~1000 Da;肽段长度的范围为5~100个氨基酸;固定修饰为半胱氨酸还原烷基化(carbamidomethyl [C]);可变修饰为甲硫氨酸氧化(oxidation [M])、蛋白质N端乙酰化(acetyl [protein N-term]);肽段谱图匹配错误发现率(FDR)≤5%。

## 2 结果与讨论

### 2.1 标准蛋白质镜像酶正交酶切产物的交联质谱分析

2.1.1 基于镜像酶切的牛血清白蛋白交联位点鉴定覆盖度

以BS^3^作为交联剂对牛血清白蛋白单一模型蛋白体系进行化学交联,并将交联样品分别采用胰蛋白酶及胰蛋白酶镜像酶进行酶解,经过质谱鉴定及数据分析,共得到了291对非冗余的交联位点信息(见附表1,详见 http://www.chrom-China.com)。

将所鉴定的交联位点信息与牛血清白蛋白的晶体结构(PDB: 3V03)进行映射,如图1所示,两种酶切方式鉴定到的交联位点存在一定互补性,初步表明基于镜像酶正交酶切的交联质谱分析策略能提高牛血清白蛋白交联位点的鉴定数目。

**图1:**
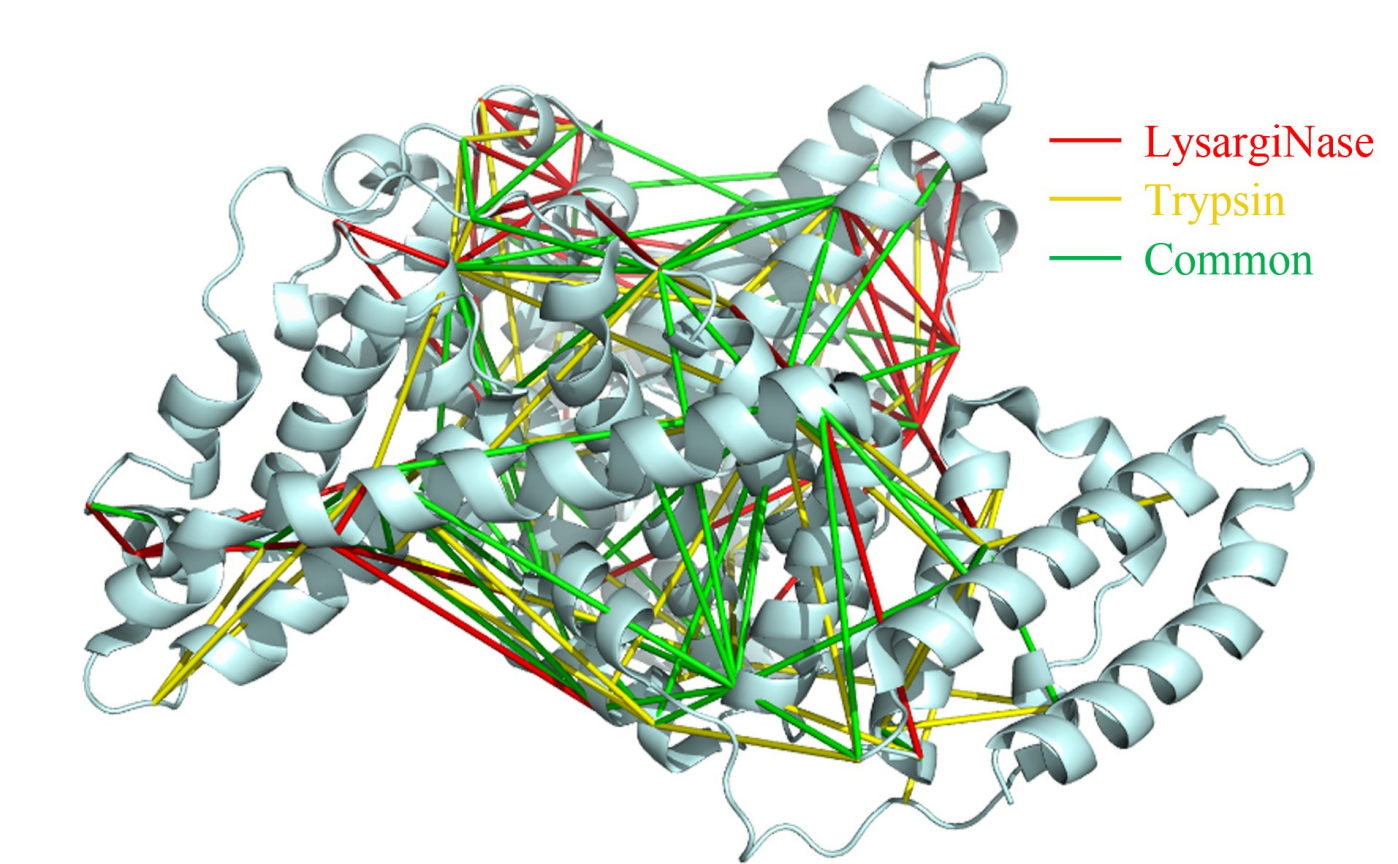
胰蛋白酶与LysargiNase酶解样品的交联位点在牛血清 白蛋白晶体结构(PDB: 3V03)的映射

如图2a所示,胰蛋白酶酶解鉴定到的交联位点的38%(82/216)被LysargiNase共同鉴定;此外,有75对交联位点被LysargiNase补充鉴定,占胰蛋白酶鉴定总数35%(75/216)。表明LysargiNase与胰蛋白酶酶解产生的交联肽段具有优异的互补性。

**图2:**
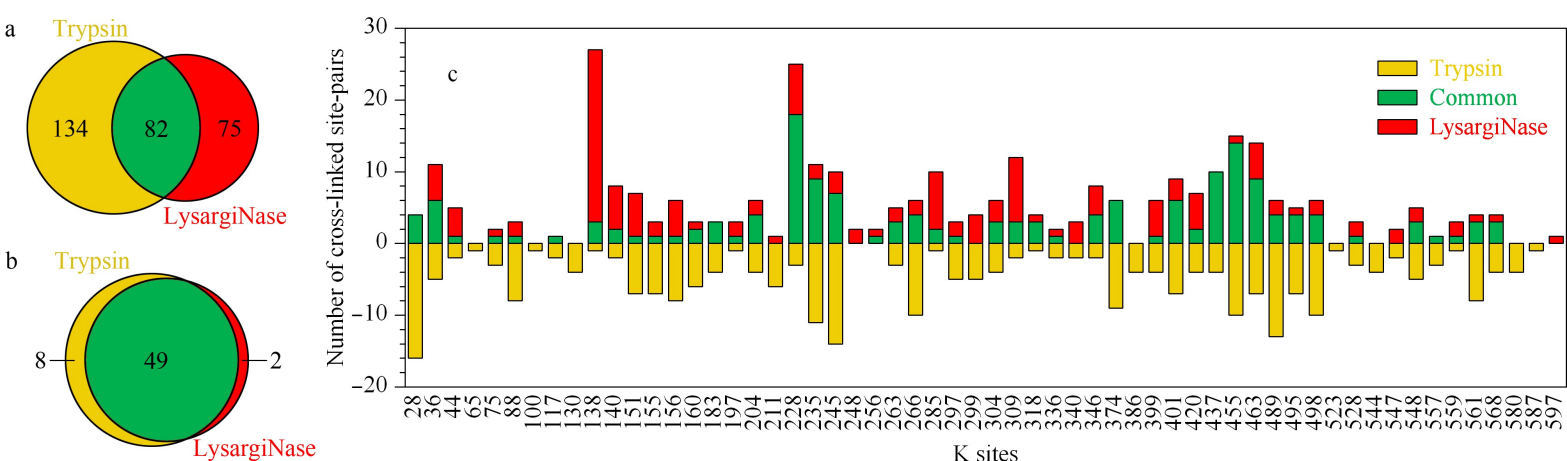
LysargiNase与胰蛋白酶酶解样品的交联位点对及单一交联位点的互补性

对两种酶切方式鉴定到的交联位点的互补性进行分析,统计交联位点对中的单一交联位点的鉴定情况,发现LysargiNase与胰蛋白酶对单一交联位点的鉴定能力并没有明显差别,83%(49/59)的位点被二者共同鉴定到(见图2b)。然而,单一交联位点在不同酶切方式所产生的交联位点对的鉴定能力不同(见图2c)。上述结果提示,两种酶切方式产生的交联位点对的互补性很可能源于质谱鉴定过程中对于二维交联肽段与线性常规肽段的不同信号响应。由于交联肽段较长,其碎片离子响应相对较差,LysargiNase较胰蛋白酶酶切虽然产生肽段的长度相当,但是由于二者酶切原理不同,K/R分别处在交联肽段的N端和C端,使得产生的交联肽段的质谱碎裂能力与响应不同,因此LysargiNase与胰蛋白酶联合正交酶切的方法,较胰蛋白酶酶解方法,能显著提高交联肽段的鉴定覆盖度。

2.1.2 基于镜像酶切的牛血清白蛋白交联位点鉴定准确度

实验对胰蛋白酶与LysargiNase两种酶切方式共同鉴定到的交联位点对应肽段的谱图质量进行了考察,以-lg(E-value)值作为打分标准,分值越高对应的谱图鉴定的准确度越高,反之准确度越低,根据共同鉴定的位点对应的-lg[E-value_(LysargiNase)_/E-value_(trypsin)_]考察镜像酶正交酶切对质谱鉴定准确度的影响。如图3所示,在二者共同鉴定的交联位点对中,32%(25/78)的交联位点对在LysargiNase的酶切结果中获得了比胰蛋白酶酶切结果更高的谱图质量得分,初步显示了镜像酶正交酶切在提高交联位点质谱鉴定准确度上的能力。

**图3:**
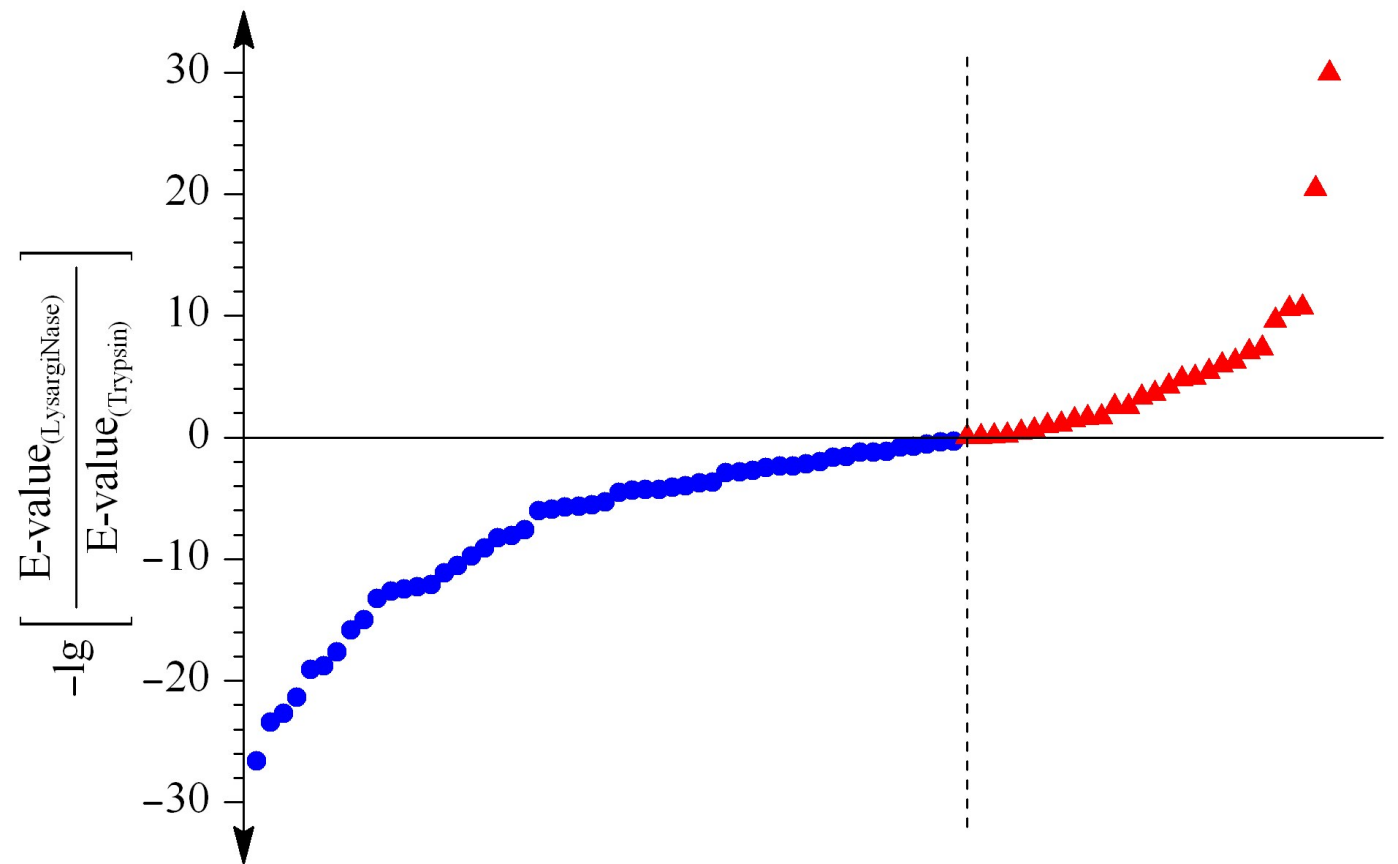
LysargiNase与胰蛋白酶酶解样品共同得到的交联位点鉴定打分比较

交联肽段是由交联剂共价连接的两条肽段(*α*/*β*)组成的,与普通肽段相比,其长度更长,理化性质更加复杂,若无法产生足够的碎片离子(b/y),则无法实现对交联位点的准确鉴定。通过脚本处理搜库结果文件,输出碎片离子鉴定信息,并统计了*α*-肽段与*β*-肽段上b^+/++^及y^+/++^碎片离子的鉴定数目,如图4所示,由LysargiNase酶切产生的交联肽段主要以b^+/++^离子碎片为主,*α*-肽段的b^+/++^离子碎片数目的平均值高于*β*-肽段的y^+/++^离子,而y^+/++^离子在*α*-肽段与*β*-肽段的碎片数目基本相当;胰蛋白酶酶解的交联肽段以y^+/++^离子为主,*α*-和*β*-肽段中y^+/++^和b^+/++^离子碎片数目相当。该结果从碎片离子的鉴定数目上初步显示了两种酶切方式产生的交联肽段在质谱检测响应行为上的互补性。

**图4:**
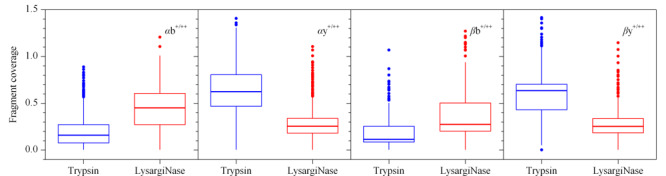
b^+/++^与y^+/++^离子碎片分别在*α*/*β*-肽段的碎片覆盖度

为了进一步考察两种酶切方式鉴定到交联肽段的谱图质量,即交联肽段与谱图匹配的准确度,我们以胰蛋白酶酶解鉴定的交联肽段的-lg(E-value)分值作为标准划分了低、中、高3个打分区间,分别为[0, 4)、[4, 8)、[8, ∞)。如图5所示,对于低打分区间(见图5a), 与胰蛋白酶相比,LysargiNase酶解所得到的交联肽段的谱图质量得到了显著提高,不仅b/y离子的匹配数目增加,其连续性也得到了提高;对于中、高打分区间内(见图5b和5c), LysargiNase的谱图质量提升效果同样被证明。因此,LysargiNase较胰蛋白酶酶解,通过提高b^+/++^离子检测效率,能够有效增加不同长度交联肽段在质谱检测中碎片离子的鉴定数目和连续性,提高肽段序列的谱图匹配质量,进而提升交联肽段的质谱鉴定准确度。

**图5:**
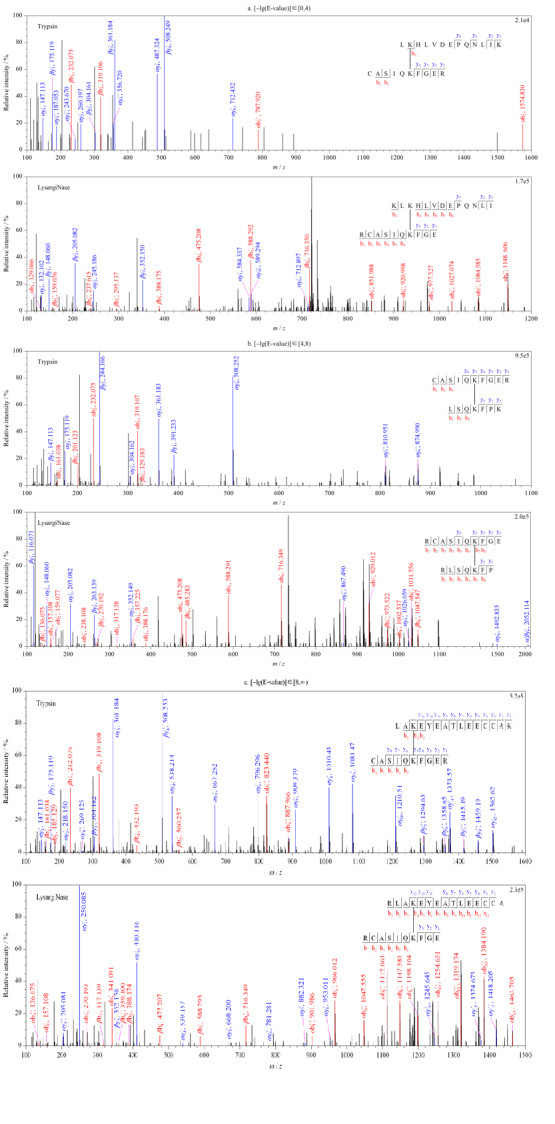
LysargiNase与胰蛋白酶酶解的交联肽段质谱图

### 2.2 复杂交联样品镜像酶正交酶切产物的交联质谱分析

2.2.1 基于镜像酶切的大肠杆菌蛋白复合物交联位点鉴定覆盖度

实验进而以大肠杆菌裂解液为样品,BS^3^为交联试剂考察镜像酶正交酶切策略对于复杂样品中蛋白质复合物交联信息的解析能力。

如附图1所示,采用镜像酶正交酶切共鉴定到726对交联位点,较单一胰蛋白酶酶切交联位点数目提高了16%(102/624);此外,有172对交联位点被LysargiNase与胰蛋白酶共同鉴定。具体的交联位点信息见附表2。总体来看,LysargiNase酶解样品交联位点的鉴定总数明显低于胰蛋白酶酶切样品,可能的原因包括以下3个方面:(1)由于交联反应主要发生于蛋白质表面,所产生的交联肽段的丰度较低,在质谱检测过程中存在随机性,尤其对于复杂样品表现得更为明显^[23]^; (2)胰蛋白酶作为蛋白质组学研究中最常用的蛋白酶,可以高效、特异地切割赖氨酸和精氨酸的C端。尽管LysargiNase被证明具有较高的酶活性和酶切特异性,其酶活较胰蛋白酶相对较低^[20]^,产生LysargiNase酶切的交联肽段由于长度过长而难以被质谱检测,尤其是在复杂样品的应用中^[20,24,25]^; (3)由胰蛋白酶酶切的交联肽段除N端外,由于酶切发生在K/R的C端,所以电荷在N端与C端都有分布,而LysargiNase的酶切位点在K/R的N端,电荷主要分布在N端,肽段整体的带电性略低于胰蛋白酶酶切的肽段,导致LysargiNase酶切的肽段在质谱中离子化效率及碎裂效率相对低于胰蛋白酶酶切产生的肽段,最终降低了LysargiNase酶切的交联肽段在质谱中的检出率。因此,上述因素制约了LysargiNase酶切的交联肽段的质谱鉴定能力,从而导致其鉴定的交联位点数目少于胰蛋白酶。

2.2.2 基于镜像酶切的大肠杆菌蛋白复合物交联位点鉴定准确度

对二者共同鉴定到的交联位点的最大-lg(E-value)值进行统计,计算各交联位点的-lg[E-value_(LysargiNase)_/E-value_(trypsin)_],用于评价LysargiNase酶切对于胰蛋白酶酶切交联位点鉴定准确度的贡献,结果显示35%(48/137)的交联位点获得了更高的鉴定得分值(见附图2)。通过考察*α*-肽段与*β*-肽段上b^+/++^及y^+/++^碎片离子的检测覆盖度,再次证实了该策略是基于交联肽段碎片离子匹配及质谱碎裂行为上的镜像互补实现了对谱图质量的提高(见附图3)。对于两种酶切产生的交联肽段的谱图进行比较,进一步证明了LysargiNase无论在低、中、高打分区间([0, 4)、[4, 8)、[8, ∞)),均可以通过提高交联肽段的碎片离子鉴定数目和连续性达到谱图质量提升的效果(见附图4),从而实现复杂蛋白体系中交联肽段的高可信度质谱鉴定。

### 2.3 大肠杆菌蛋白质复合物交联鉴定结果分析

综合LysargiNase与胰蛋白酶两种酶切方式所鉴定的大肠杆菌中交联位点信息,我们共鉴定到29对蛋白质分子间的相互作用,对应119对交联位点(见图6a),以及242个蛋白质分子内的607对交联位点信息。在已鉴定的29对蛋白质相互作用中,12对已被大肠杆菌整合蛋白质相互作用数据库(http://www.bacteriome.org)所报道^[26]^,此外,有10对相互作用与已报道的化学交联蛋白互作数据相吻合^[13]^,证实了本方法所获得的分子间交联信息的可靠性。另外,还有7组蛋白质间相互作用未见报道,有望为大肠杆菌中的蛋白质互作谱图的全面绘制提供重要的数据参考。

**图6:**
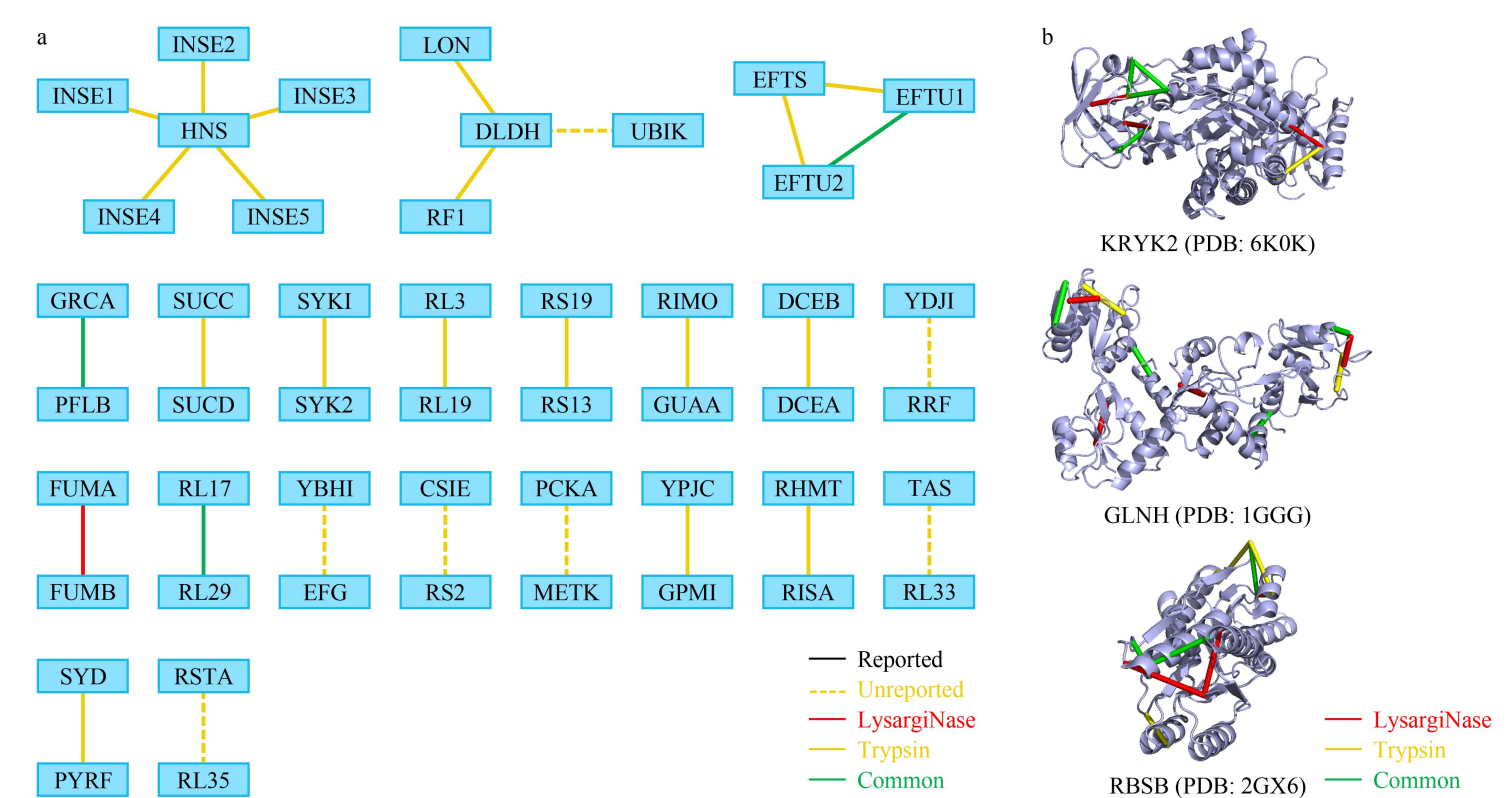
大肠杆菌样品中LysargiNase与胰蛋白酶酶切鉴定蛋白质复合物信息互补性

蛋白质复合物在执行其功能的过程中依赖蛋白质分子的结构调控蛋白质相互作用的动态组装,交联位点的高准确度和高覆盖度鉴定对于揭示蛋白质复合物功能具有重要的作用。我们对获得的大肠杆菌体系中242个蛋白质的607个分子内交联位点信息进行分析。其中,由胰蛋白酶单独鉴定到的分子内交联位点数目为364个,由胰蛋白酶与LysargiNase共同鉴定到的分子内交联位点数目为149个,由LysargiNase单独鉴定到的为94个。对于共同鉴定到的蛋白质,其交联位点鉴定数目较单一胰蛋白酶酶切,平均提高了34%(见附表3),表明镜像酶正交酶切策略能显著提高蛋白质结构交联位点的鉴定覆盖度。进一步,我们以丙酮酸激酶II(KPYK2)、谷氨酰胺结合周质蛋白(GLNH)、核糖导入结合蛋白(RBSB)为例,说明该方法在蛋白质复合物结构位点分析中的优势(见图6b,具体交联位点信息见附表4)。

丙酮酸激酶II蛋白在糖酵解的最后一步催化丙酮酸的形成,在生理条件下是不可逆的,对于糖酵解第二部分中代谢通量的控制至关重要。通过LysargiNase与胰蛋白酶联用的镜像酶正交酶切策略我们获得了与KPYK2相关的8组交联位点信息,将交联位点信息映射到先前发布的KPYK2(PDB: 6K0K)结构上,两个交联位点之间的直线距离均在BS^3^的最大交联距离限制之内(2.4 nm),满足结构兼容性要求,证实了交联数据的可靠性。其中,由胰蛋白酶酶解样品单独提供的交联位点信息有1组,有3组交联位点信息只被LysargiNase酶解的样品鉴定到,有4组交联位点信息被胰蛋白酶与LysargiNase共同鉴定,证明了该策略具有提高交联位点信息准确度及覆盖度的能力。

谷氨酰胺结合周质蛋白在谷氨酰胺转运系统中发挥作用,对于谷氨酰胺通透酶活性是必不可少的,通过镜像酶正交酶切策略,我们获得了7组交联位点数据,其中由胰蛋白酶酶解的交联样品单独提供的交联位点有1组,而由LysargiNase酶解的样品单独提供的有3组,两种酶解样品共同提供的位点信息有3组。将交联信息映射到先前发布的GLNH的晶体结构上(PDB: 1GGG),有5组交联位点信息能够与该晶体结构匹配,且各交联位点之间的直线距离均小于交联剂最大交联距离约束,证实了交联结果具有高可靠性,未匹配的交联信息有可能完善GLNH的结构分析。

核糖导入结合蛋白的交联质谱分析结果也同样证明了该方法对鉴定覆盖度的提高。该蛋白质为ABC转运蛋白复合物RbsABC的一部分,涉及核糖的结合与引入,同时也作为趋化性的主要化学感受器。通过镜像酶正交酶切策略,我们总计获得11组交联位点数据,其中由胰蛋白酶酶解单独提供的交联位点有5组,由胰蛋白酶与LysargiNase共同提供的有3组,由LysargiNase单独提供的有3组;并且得到的交联位点在晶体结构(PDB: 2GX6)中的直线距离均符合交联剂的距离约束,再次证实了该方法得到的交联位点的可信性。

## 3 结论

本文提出了一种基于LysargiNase与胰蛋白酶镜像酶正交酶切的化学交联质谱技术。对牛血清白蛋白和大肠杆菌裂解液蛋白的分析结果表明,该方法从酶切的角度出发,以镜像互补的方式显著增加了肽段特征碎片离子的鉴定数目和匹配连续性,提升了交联肽段与谱图匹配的准确度,提高了交联位点鉴定准确度及覆盖度,有望为实现规模化的蛋白质复合物精准解析提供新思路。
